# Physical Characterization of Cementitious Materials on Casting and Placing Process

**DOI:** 10.3390/ma7043049

**Published:** 2014-04-15

**Authors:** Hong Jae Yim, Jae Hong Kim

**Affiliations:** 1 Department of Civil and Environmental Engineering, Korea Advanced Institute of Science and Technology, Daejeon 305-701, Korea; E-Mail: yimhongjae@gmail.com; 2 School of Urban and Environmental Engineering, Ulsan National Institute of Science and Technology, Ulsan 689-798, Korea

**Keywords:** cement paste, particle size, coagulation, flocculation, laser backscattering

## Abstract

Coagulation of cement particles is an inevitable phenomenon of fresh cement-based materials undergoing solidification. Coagulation can be classified into two types, reversible flocculation and irreversible coagulation, wherein microstructural change affects the rheological properties, including shear thinning and thixotropy, and the hydration process. This paper attempts to measure the mechanical property and the coagulation of cement particles according to the mix proportions of cement paste. Experimental setups were proposed for two different types of coagulations using a laser backscattering instrument. Volume fraction and size distribution of coagulated particles were obtained, and their variations were discussed. From the obtained results the microstructural buildup of freshly mixed cement pastes can be divided into three categories: permanent coagulation and strong and weak flocculation.

## 1. Introduction

Cementitious materials, including portland cement, pozzolans and granulated slag, are extensively consumed for building infrastructures these days. The cementitious material provides binding properties to compose bulk engineering materials, of which the majority of the volume is occupied by inert fillers, such as natural aggregates. Its binding property is manifested by the hydration of cementitious material, and its aqueous suspension temporarily exists as a precursory state. The time of suspension is called the dormant period because the chemical reaction to produce binding compounds momentarily pauses before its activation. In the case of portland cement, the time for which the powder-type cementitious material can be suspended is more than 1 h, and the solidification of suspension is then followed with the passage of time. For engineering practices, the period of suspension is critical for the casting and placing process of the cement-based materials, which is the only time which allows us to cast products. Therefore understanding of the suspension of cementitious materials enhances the casting and placing performance of cementitious composites and, consequently, secures the quality of infrastructure materials.

One of the most principal phenomena in the suspension of cementitious materials is coagulation, a process of dispersed particles growing in the form of clusters [1]. Pulverized cement in water involves combination and adhesion of cement particles due to potential energy of van der Waals attraction [2], which results in coagulation, solid percolation in the suspension, and, finally, solidification with cement hydration. Notably, such a change of the fresh microstructure affects cement hydration and the rheological properties, including shear thinning and thixotropy [3].

The coagulation can be classified into two types, reversible or irreversible phenomenon. The reversible coagulation is generally referred to as flocculation, and flocculated particles can be separated by an applied external power larger than their flocculation strength. When fresh cement-based material is under high shear flow and high pressure, such as in the case of pumping and vibrated consolidation, the flocculated cement particles are broken in practice. In contrast, irreversible coagulation leaves permanent coagulates that cannot be broken by any physical agitation. The permanent coagulation is due to quick setting of cementitious materials, which is related to the monosulfate and ettringite formation with the hydration of aluminate (3CaO·Al_2_O_3_). Even in the dormant period of lime-silica compounds (3CaO·SiO_2_ or 2CaO·SiO_2_), holding a large majority in cementitious materials, the aluminate hydration activates on the coagulated particles and tightly ties them. Its amount of reactive products is small but critical to control the early-age stiffening of suspension, which decreases its consistency.

The kinematics of cement particles in aqueous suspension can be monitored using a laser backscattering instrument. Cement particles and clusters reflect the incident laser and the delay intensity of the backscattered laser indicates the chord length of the particles in suspension. Its *in situ* applications in a reactor have been widely reported in the field of chemical engineering [4–9]. For a neat cement paste, it was found that the flocculation is affected by the alkalis/alumina content, and nanoclay is one of the most influential admixtures with respect to the flocculation rate [10,11]. Furthermore, the effect of the flocculant on the fiber-cement suspension was observed during the manufacturing process [12–14].

Previous studies on cement-based materials show the possibility of quantitative measurement of cement coagulation, where only the mean size (μ) of cement flocs was monitored to reveal the effects of admixtures and chemical contents. The amount of coagulation and their size distribution have not been quantitatively monitored, even though researchers presume that coagulation increases with the passage of time. This paper attempts to measure the coagulation of portland cement particles and the effect of mix proportions is also investigated. Two different types of coagulation, permanent coagulation and flocculation, are separately measured with two different setups proposed in the study. From the obtained results, their volume fraction (ϕ) and size distribution are obtained and the microstructural buildup of freshly mixed cement pastes is revealed.

## 2. Experiment

### 2.1. Sample Preparation

A total of five cement paste mixes were prepared with ordinary Type I portland cement. The specific gravity and specific surface determined by the Blaine method are 3.14 and 3320 cm^2^/g, respectively. Table 1 shows the oxide composition of the used powders.

The mix proportion of each sample to obtain a 30 mL volume is described in Table 2, where two comparison sets are found according to the water-to-cement ratio (*w*/cm) and the dosage of high-range water-reducing admixture (HRWRA). The *w*/cm by mass was 40%, 30%, and 60% for mixes C1, C4, and C5, respectively. The effect of HRWRA was also investigated in mixtures having a water-to-powder ratio of 40% (mixes C1, C2, and C3). The used HRWRA was a polycarboxylate-based admixture with a solid content of 30% by mass (ADVA 128, W. R. Grace & Co., Columbia, MD, USA). The HRWRA dosage was 0.2% and 0.35% of cement mass, which are, respectively, labeled C2 and C3, while no HRWRA is mixed for C1. All samples were produced according to the following mixing procedure: (1) hand-mixing for 2 min; (2) scraping the mixing beaker (100 mL volume) for 1 min; and (3) hand-mixing again for 2 min. The room temperature (22 °C) and humidity (50%) were maintained during the mixing and following experiment.

### 2.2. Flow Curves Determination

The flow curves of the samples were measured to determine their rheological properties. A commercialized rheometer (HAAKE MARS III, Thermo Fisher Scientific Inc., Waltham, MA, USA) was used. The measuring geometry of the rheometer was parallel plates with a diameter of 35 mm and the gap between the plates was set to 1 mm. The rheological measurement was obtained immediately after the mixing was finished. The protocol for the measurement followed ASTM C1749 [15]. After 1 min pre-shearing at 100 s^−1^, the step of strain rate was decreased as 80 s^−1^, 60 s^−1^, 40 s^−1^, 20 s^−1^, 10 s^−1^, 5 s^−1^, 1 s^−1^, 0.5 s^−1^, and 0.1 s^−1^, sequentially. The shear stress was measured for 20 s at each step, and the final shear stress was obtained when the value became stable. The measured flow curves were described in the next section. Each 30 mL sample was encapsulated to prevent water evaporation and maintained in a static condition. A small amount of the encapsulated sample was taken and experimented on in series. Different experimental procedures were proposed depending on the phenomenon to be characterized: (1) flocculation and (2) permanent coagulation. Details are described in the next section.

### 2.3. Laser Backscattering Instrument

Monitoring dimensional information of particles in a suspension was accomplished with a laser backscattering instrument (ORM, optical-back reflectance measurement, Sequip GmbH, Düsseldorf, Germany). Through the ORM probe, a laser beam with an intensity of 3 mW is emitted. The laser beam is dynamically focused on the front head, which is covered with Sapphire glass, having a diameter of 17.8 mm. The dynamic focus follows a three-dimensional elliptical orbit, where it makes an 8.5 mm-diameter circle horizontally and also moves in a normal direction, dynamically set at 40 μm to 125 μm above the window. The laser optic system obtains the scattered time of the laser signals and then determines the length of particles and their population. As a result, the number and length distribution of the particles are measured within a range of 0.5 μm to 1200 μm. The resolution is 0.5 μm. The unweighted size distribution can also be converted into the area or volume fraction [8].

### 2.4. Experiment for Permanent Coagulation

To investigate permanent coagulates in cement pastes it is necessary to first eliminate breakable (reversible) particles in the samples. Each sample was diluted with isopropyl alcohol as follows: 4 mL-paste in 400 mL-alcohol (1% volume fraction). The use of alcohol prevents cement hydration and particle interaction. Alcohol is also expected to help flocs fragment quickly. The diluted sample was stirred for 5 min at 900 RPM to break the clusters. Here, it is assumed that the flocculation strength is less than the shear energy applied at 900 RPM. Successive stirring was carried out at 600 RPM, where the particle size distribution was measured after the flow in a beaker became stable. The time to obtain a stable flow and, thus, consistent measurement was less than 10 min. In the subsequent 10 min period, the size distribution of permanent coagulates was measured with an increment of 1 min and the average value is reported.

**Figure 1.**
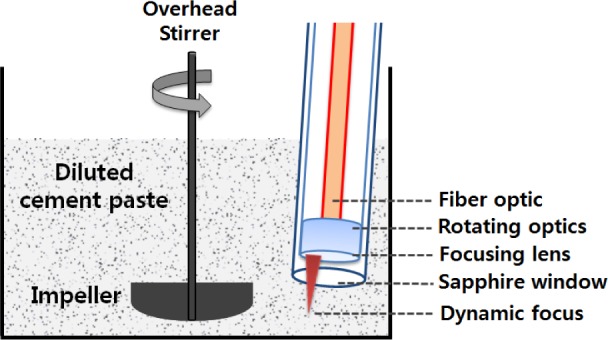
Top-down setup for permanent flocculates.

Figure 1 shows the conventional top-down setup and the diluted sample is placed in a 1 L beaker (110 mm diameter by 150 mm height). An impeller, tilted at 20° to avoid turbulence of the suspension, is located 10 mm above the bottom of the beaker. A half-moon axial impeller is used in the study, which is composed of two collapsible blades made with PTFE and its size is 65 mm × 18 mm × 3 mm. According to the agitating procedure described above, the diluted sample to be tested is agitated with an overhead stirrer (WiseStir, HT-120DX, Wertheim, Germany).

### 2.5. Experiment for Flocculation

Flocculation strength is various in cement paste. At a certain shear rate, some clusters, having a small flocculation strength, are broken while others, having a large flocculation strength, are not. At higher agitating speeds, more clusters are broken into small particles. On the other hand, the particles that partially deflocculated are too large (heavy) to float up at low-speed agitation. Increase of the agitating speed causes both deflocculation and floating large particles, which results in decreased particle size and quantity (count) of particles at the bottom of suspension. In order to capture these phenomena, a bottom-up setup for water dilution was used. A 1.5 mL sample was diluted in 150 mL of water (1% volume fraction) for measurement. The deflocculation ratio was measured while the agitating speed was gradually increased from 500 to 900 RPM in increments of 100 RPM. At each RPM, the number and size distribution of particles were measured during 10 s to stabilize the bottom-up setup for measurement of flocculation.

**Figure 2.**
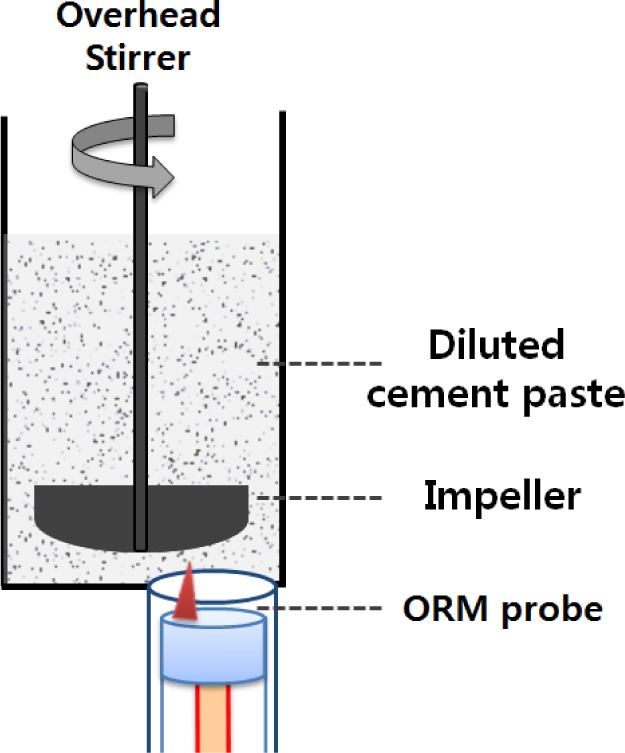
Bottom-up setup for flocculates.

Figure 2 schematically shows the experimental setup where the ORM probe is placed bottom-up to a diluted sample. The diluted sample is placed in a fiber-pipe container having a diameter of 53 mm. The ORM probe is located at the bottom of the container and its axis is aside from the central axis of the container. The 150-mL diluted sample fills the container and the sample height reaches 69 mm. After the sample is placed, an impeller is put at the center of the bottom. The measurement procedure is then followed with the same impeller and agitator.

## 3. Results and Discussion

### 3.1. Flow Curves of the Samples

**Figure 3.**
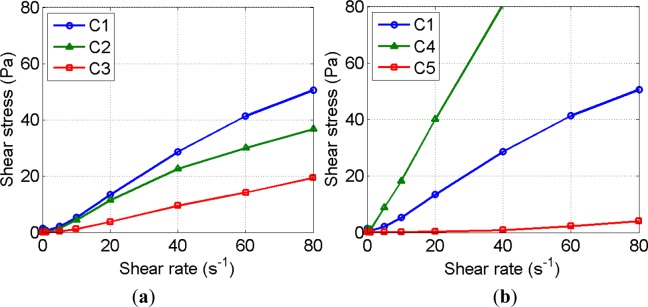
Flow curves of the samples according to the (**a**) different HRWRA dosage and (**b**) different *w*/cm.

Figure 3 shows the data points measured at each step, according to the protocol and their trend lines are also plotted. Each flow curve is the average of three measurements with their replicated samples. The yield stress of all samples is not significant and their flow curves follow a power law function. The use of HRWRA or higher water-to-cement ratio decreases the viscosity, as expected.

### 3.2. Raw Powder

**Figure 4.**
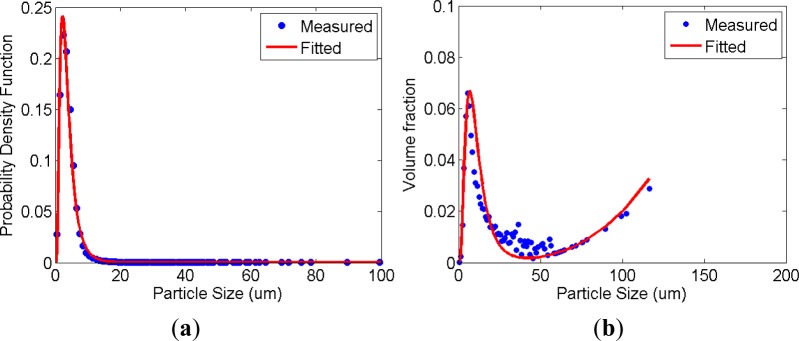
Size distribution of cement powder: (**a**) the measured number distribution and (**b**) the converted volume distribution.

Prior to the measurement of cement paste, the size distributions of raw cement powder were measured. Alcoholic dilution and the top-down conventional setup for permanent coagulation were used to measure the diameters (*d*) of each grain. Based on the measurement data, the probability distribution (number distribution) of grain diameter is achieved. The volume fraction of the powder can be calculated by multiplying the probability distribution by the cube of diameter *d*^3^. While the probability distribution represents the counts at intervals of their diameter, the volume fraction describes the volume fraction at each interval. The distribution of solid volume fraction explains the packing and rheology of the pastes. Figure 4 shows the measured probability distribution and its volume fraction. The volume fraction of the cement powder hits the local minimum at about 34 μm and it rises. In contrast, the particle counts of which diameter is larger than 10 μm are negligible in the probability distribution. Even a very small amount of large particles highly influences the volume fraction and packing. In the current data, the particles larger than 10 μm are due to cement agglomeration and it is natural in practical storage of cement powder.

In this research, the grain diameter of raw cement powder is assumed to follow a lognormal distribution, which is a common type of probability distribution for raw cement powder size. In other words:

$$d\sim \text{LN}(\mu_{c},\sigma_{c})$$ (1)

where *d* is the grain diameter and LN(μ_c_, σ_c_) denotes the lognormal distribution with mean μ_c_ and standard deviation σ_c_. The powder grains smaller than 10 μm are less sensitive to the large particles agglomerated. Therefore, their mean and standard deviation within the range were evaluated as reported by Table 3 (raw grains).

The large-particles tail in the volume fraction is apt for investigating agglomeration. The assumption that the agglomeration does not change the total volume of solid particles provides a complementary volumetric relation on the agglomeration. This corresponds to the fractal dimension of 3, where the floc porosity is constant during coagulation [16]. In such a situation, the decrease in the volume of raw cement powders (1 − ϕ_a_) is the same as the increase in the total volume of their agglomerates (1 − ϕ_a_). Another lognormal distribution for large agglomerates is introduced to Equation (1) for describing the probability density function of grain diameter:

$$d\sim \phi_{\text{a}}\cdot \text{LN}(\mu_{\text{a}},\sigma_{\text{a}})+(1-\phi_{\text{a}})\cdot \text{LN}(\mu_{\text{c}},\sigma_{\text{c}})$$ (2)

where *d* is the grain diameter, ϕ_a_ is the total volume fraction, and μ_a_ and σ_a_ are the mean and standard deviation of the agglomerates diameter. The probability distribution in Equation (2) is a mixture distribution which is the weighted combination of two probability distributions. Equation (2) multiplied by the cube of diameter *d*^3^ is fitted to the volume fraction using *nlinfit*, a MATLAB^®^ function for nonlinear regression analysis so that the least square error between the measured data and the fitted probability distribution was minimized. The three unknown parameters of the agglomerates were consequently obtained. Its total volume fraction (ϕ_p_) is 0.012 and its mean (μ_a_) and standard deviation (σ_a_) are reported in Table 3 (agglomerates). In addition, in Figure 4, the fitted curve is shown by red lines. In the next section, it will be shown that the distribution of permanent coagulation and flocculation has a greater portion of the tail of large particles.

### 3.3. Permanent Coagulation

**Figure 5.**
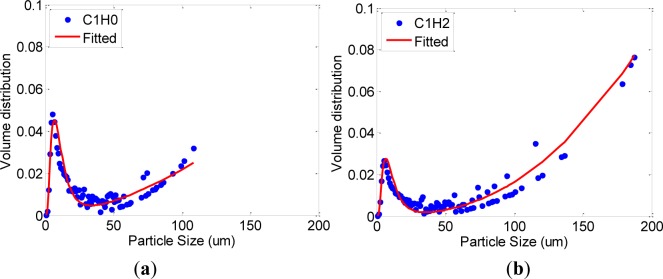
Volume distributions of mix C1 at (**a**) 0 h and (**b**) 2 h after mixing.

Coagulation accelerates when the cement paste samples in air are at rest. Sometimes, it is called agglomeration. The dimensional information of permanent coagulates was measured with a top-down conventional setup with alcoholic dilution. Fitting the volume fraction provides quantitative information on the permanent coagulation. Figure 5 shows the change in the volume fraction of mix C1, where the size of permanent coagulates is increasing with the passage of time at rest. The volume distribution at 4 h is very similar with the 2-h-measurement, except that the small size peak (at around 10 μm) is slightly decreased and the large size tail is more tip-tilted.

The assumption of the complementary volumetric relation is also applied on the coagulation. Another lognormal distribution for permanent coagulates is here introduced:

$$d_{p}\sim \phi_{p}\cdot \text{LN}\left(\mu_{p},\sigma_{p}\right)+(1-\phi_{p})\cdot \text{LN}\left(\mu_{c},\sigma_{c}\right)$$ (3)

where *d*_p_ is the grain diameter after coagulation, *ϕ*_p_ the total volume fraction, and μ_p_ and σ_p_ are the mean and standard deviation of the permanent coagulates diameter. Then, it is fitted again with *nlinfit*, a MATLAB^®^ function. Table 4 reports the increase in the volume fraction of permanent coagulates. A threshold of *w*/cm exists at which a mix provides a consistent amount of permanent coagulation. In mixes C1 (*w*/cm 40%) and C4 (*w*/cm 30%) the respective amounts of permanent coagulation for 4 h were 18% and 14%, respectively, but C5 (*w*/cm 60%) showed a value of about 5%. Packing cement particles under such a low-threshold population provides a similar rate of permanent coagulation but decreasing the concentration of cement particles reduces the permanent coagulation.

Incorporating HRWRA reduces and decelerates permanent coagulation as found in mix C3. HRWRA is a polymer to increase the consistency of cement suspension when it is added in a very small amount (approximately 0.30% to 0.45% by weight of cement). It is composed of a hydrocarbon backbone having multiple polar groups and then easily attaches to cement particles. As a result, it causes deflocculation of cement particles and makes them hydrophilic. The deflocculation monitored in the superplasticized mixes, C2 and C3, will be discussed in the next section and the deflocculation also affects the permanent coagulation (retarding) as shown by high-dosage HRWRA mix C3.

**Figure 6.**
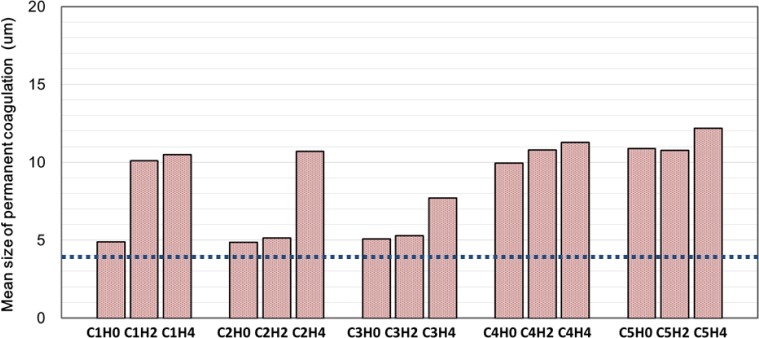
Mean size of permanent coagulation (the dotted line is the mean of cement powder (μ_c_), 3.84 μm).

The mean sizes of permanent coagulates are shown in Figure 6, where the values are higher than that of raw cement powder (3.84 μm) but sometimes smaller than that of agglomerates (7.74 μm). The mixing process is thought to break the weak agglomeration and rebuild strong flocculation. However, their variation with time and the effect of mix proportion are not significant: within 4 h, the maximum increase of mean size is only 7 μm from 5 μm. This increase is not significant compared to the flocculation discussed in the next section.

### 3.4. Flocculation

Dimensional information of flocculation is not easy to obtain because diluting a sample promptly causes deflocculation. Nevertheless, reversibility of flocculation allows us to use an indirect method: Measurement of deflocculation inversely indicates the rate of flocculation. The quantity and dimension of flocs are monitored during the process of deflocculation. For this purpose, a bottom-up setup for water dilution was used. Other factors affecting the rate of deflocculation, such as diluting concentration and its volume, are kept constant. The agitation speed is the only variable.

**Figure 7.**
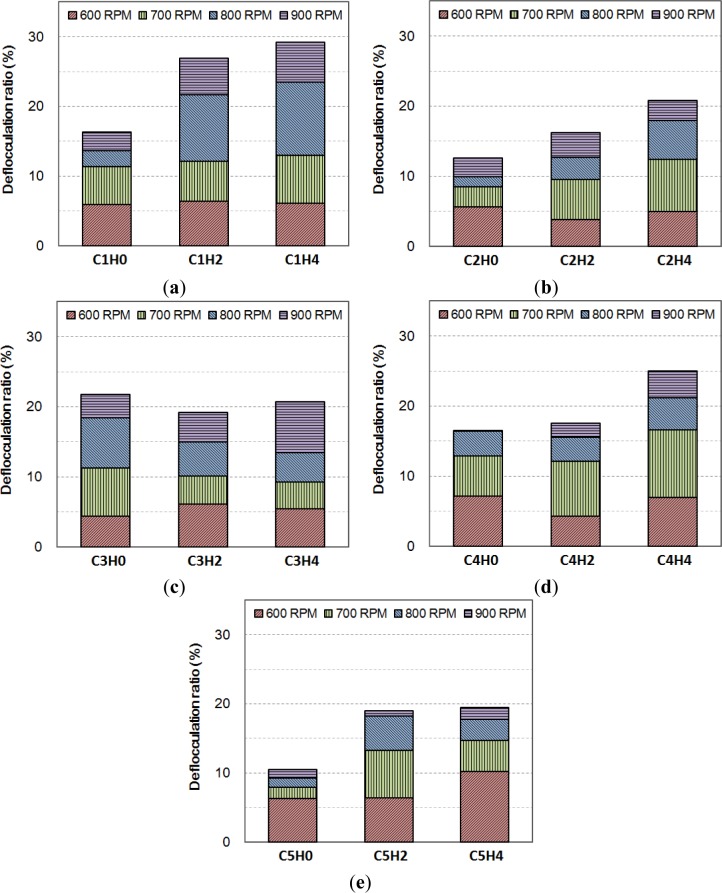
Deflocculation ratio of the cement pastes: (**a**) C1; (**b**) C2; (**c**) C3; (**d**) C4; and (**e**) C5, where the suffix H0, H2, and H4 implies the passage of time at rest.

The quantity (count) of coagulated flocs was measured and its value decreases with increasing agitating speed from 500 to 900 RPM. Floating of deflocculated small particles results in a decrease of particle quantity at the bottom. Figure 7 shows the deflocculation ratio defined by the decreased ratio of the particle quantity at the 500 RPM reference measurement. For example, for the initial measurement (labeled by the suffix H0) of mix C1, 6% deflocculation was observed with the change of 500 RPM to 600 RPM. Further increase to 700 RPM, 800 RPM, and 900 RPM causes deflocculation up to 12%, 14%, and 16%, respectively. The passage of time at rest increases the amount of strong flocculation (900-RPM measurement), which is supported by higher deflocculation ratios for a few-hour-old samples. The samples were at rest for 2 and 4 h, labeled by the suffixes H2 and H4, respectively. The only exception is mix C3 incorporating high-dosage HRWRA, where there is little change of the amount of flocculation over time and the amount is much higher than that of the others. A high dosage of HRWRA appears to restrain deflocculation during the mix process. In contrast, the deflocculation ratio of C5 (*w*/cm 60%) is lower than that of C4 (*w*/cm 30%) when measured before hydration (at H0), and a lower volume fraction of cement particles in the mix promotes deflocculation during the mixing process.

**Figure 8.**
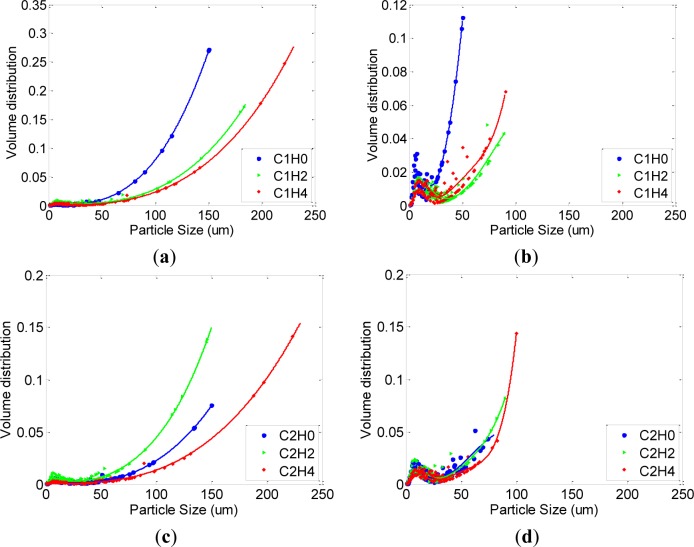

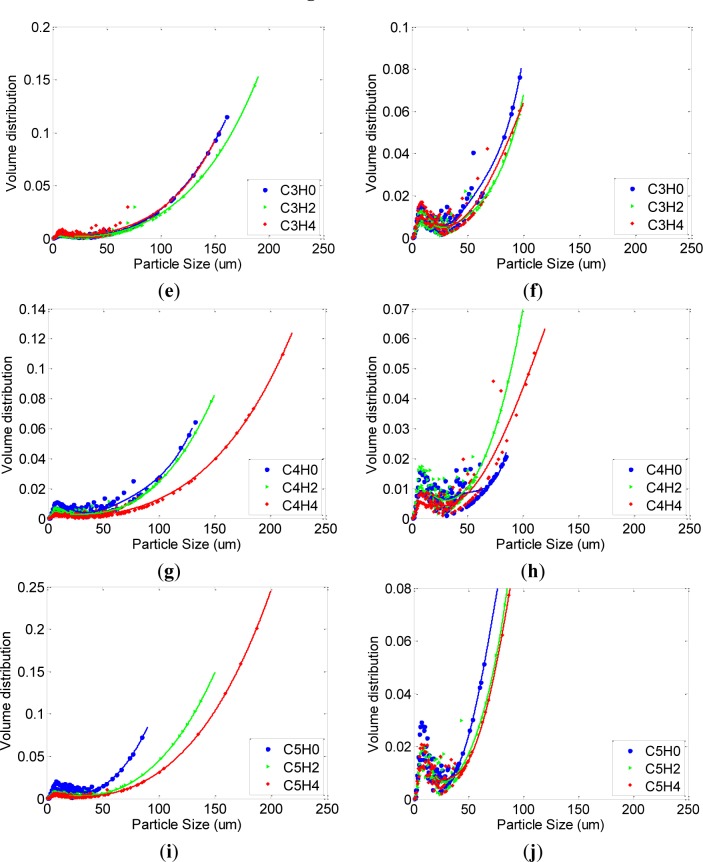
Volume distributions of mixes C1 to C5 (**a**) mix C1 at 500 RPM; (**b**) mix C1 at 900 RPM; (**c**) mix C2 at 500 RPM; (**d**) mix C2 at 900 RPM; (**e**) mix C3 at 500 RPM; (**f**) mix C3 at 900 RPM; (**g**) mix C4 at 500 RPM; (**h**) mix C4 at 900 RPM; (**i**) mix C5 at 500 RPM; (**j**) mix C5 at 900 RPM.

Their volume fractions are also shown in Figure 8, where only the measurements at 500 RPM and 900 RPM are displayed for simplicity. At the 500 RPM measurement, small particles (0–30 μm) observed in permanent flocculation (see Figure 5) are not found and large particles between 50 and 250 μm dominate the volume fraction. The large particles generally increase with the passage of time. Mix C2 was an exceptional case, and shows that the use of HRWRA can reduce the degree of flocculation as the passage of time. On the other hand, applying high-speed agitation up to 900 RPM left-squeezes the volume fraction. In particular, the population of small particles distributed at approximately 10 μm increases with 900 RPM. The loss of larger particles and increase of small particles confirms that deflocculation occurs in the measurement procedure. In addition, particles at all ages (0, 2, and 4 h) have similar size of less than 100 μm under such high-speed agitation (900 RPM). The agitation did not provide sufficient power exceeding the flocculation strength at this level, indicating that the strong flocs asymptotically approach the state of permanent coagulates. The trend of particle size becomes similar with the volume fractions of permanent coagulate shown by Figure 5, where small particles between 1 and 50 μm reappear.

In this study, it is assumed that strong flocculation is broken at 900 RPM agitation for a sample volume used in the bottom-up setup. The dimensional range of the flocculation is between 50 and 250 μm, and some samples were also observed to be in a state of permanent coagulation (see Figure 5). They are quantitatively extracted by the deflocculation ratio shown by Figure 7 and the results are summarized in Table 5. The initial measurement denoted by the suffix H0 varies among the samples even though the same mixing protocol and time were applied for all mixes. The mixing energy applied to the samples varies with their rheological properties, as shown in Figure 1, and, hence, the initial degree of flocculation depends on the mix proportion.

The *w*/cm effect is not trivial: The mix with *w*/cm 30% (C4) yielded a similar increase of strong flocculation compared to the control mix C1 (*w*/cm 40%). In particular, the initial measurements of strong flocculation are approximately identical for mixes C1 and C4 even though the viscosity of mix C4 is around twice that of mix C1 (see Figure 1). Similar to the case of permanent coagulation, a threshold of *w*/cm exists for the initial state of strong flocculation (at H0). A mix having a lower *w*/cm than the threshold value experiences a similar amount of strong flocculation: The strong flocculation is about 16% for a mix having a *w*/cm less than 40%. For those mixes, their rheology highly depends on their concentration (solid volume fraction) rather than flocculation and the mixing protocol. Increasing *w*/cm up to 60%, as in the case of mix C5, significantly reduces the strong flocculation, as expected. Less packing in a mix results in less flocculation. However, the rate of strong flocculation does not follow the effect of *w*/cm. The increases for 4 h are 13%, 8%, and 9% for mixes C1, C4, and C5, respectively. In the superplasticized mixes, C2 and C3, the initial amount of strong flocculation and the increase of strong flocculation are not bounded by mixes C1 and C5, where their viscosities were bounded by mixes C1 and C5 (see Figure 1). Mix C3, having high-dosage HRWRA, does not show an increase of strong flocculation, consistent with the status of its permanent coagulation. The rheology of those mixes is probably dependent on the particle dispersion rather than on strong flocculation. The particle dispersion would be distributed in the form of weak flocculation.

### 3.5. Microstructural Buildup

**Figure 9.**
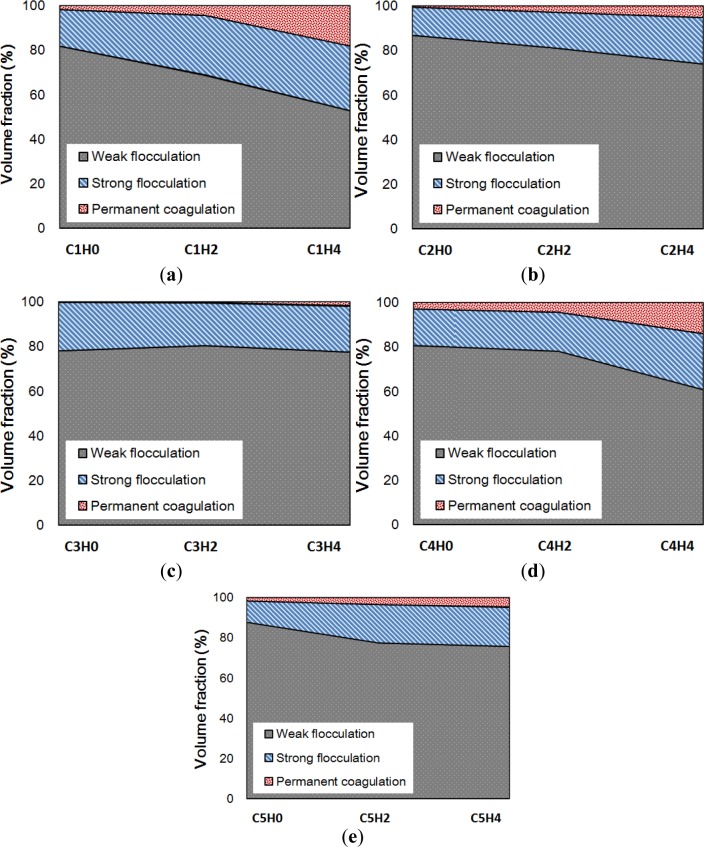
Ratio of suspended particles and its evolution according to cement hydration: (**a**) C1; (**b**) C2; (**c**) C3; (**d**) C4; and (**e**) C5.

In the previous sections, the amounts of permanent coagulation and strong flocculation, respectively, were evaluated. The remaining wet powder in a mix stays in the form of a weakly flocculated network. The weak flocculation is easily broken by external shearing, which results in thixotropy. The values listed in Tables 4 and 5 show the evolution of permanent coagulation and strong and weak flocculation, as is also shown in Figure 9.

Comparing the viscosity with the amount of coagulation makes a link between the microstructure of cement suspension and its mechanical property. As reported in Figure 1, the viscosity of cement suspension decreases with increasing *w*/cm ratio and incorporating HRWRA. The amount of permanent coagulation follows the order of viscosity but that of strong flocculation is not in order of viscosity. The rheological protocol to measure the viscosity applies descending rate of shear strain with initial preshearing, which results in break the majority of flocculation. The measured flow curve, therefore, represents the deflocculated state of suspension disregarding its thixotropy. In the deflocculated state, the mechanical property of suspension is determined by the characteristics of permanent coagulation. Such an argument for strong and weak flocculation can be made with the measurement of thixotropic behavior. Picking the thixotropy on the flow curve needs a special rheological protocol, which is beyond the scope of the paper and will be continued in future study.

## 4. Conclusions

This study investigates the mechanical property of suspension of cementitious materials. The measured viscosity is related to coagulation of cement particles and then their coagulation was monitored. Two different setups were proposed to measure the permanent coagulation and strong flocculation separately. The permanent coagulation is responsible for an increase of cement particles up to 12 μm while the mean size of the raw powder is 3.8 μm. The effect of mix proportion on the size is minimal but a certain threshold of *w*/cm exists with respect to the rate of permanent coagulation. The increase of permanent coagulation is constant for a mix produced with a lower *w*/cm that the threshold. In this study, a *w*/cm lower than 40% was considered. The threshold also applies to strong flocculation distributed between 50 and 250 μm. Initial measurement of strong flocculation is constant for the mixes with lower *w*/cm than the threshold. The use of HRWRA diminishes both permanent coagulation and strong flocculation. For a mix having high dosage coagulation was not observed. Finally, from observation of particle sizes in the cement paste, the microstructural buildup was classified into three categories: permanent coagulation and strong and weak flocculation.

## Figures and Tables

**Table 1.** Oxide composition of the cement.

Oxide	CaO	SiO_2_	Al_2_O_3_	MgO	SO_3_	Fe_2_O_3_	K_2_O	TiO_2_	Na_2_O
Percent (%)	63.6	19.2	4.8	3.8	3.7	3.0	1.1	0.3	0.2

**Table 2.** Mix proportions of prepared samples.

Label	*w*/cm (%)	Water (g)	Cement (g)	HRWRA (g)
C1	40	17	42	–
C2	40	17	42	0.084
C3	40	17	42	0.146
C4	30	15	49	–
C5	60	20	33	–

**Table 3.** Size distribution of the cement powder.

Diameter distribution, μm	Raw grains	Agglomerates
mean	3.84	7.74
standard deviation	2.33	1.59

**Table 4.** Volume fraction of permanent coagulation (%).

Time (h)	Mix C1	Mix C2	Mix C3	Mix C4	Mix C5
0	1.76	0.53	0.18	2.86	1.63
2	4.28	2.8	0.35	4.3	3.51
4	18.2	5.1	1.8	14.12	4.76

**Table 5.** Volume fraction of strong flocculation (%).

Time (h)	Mix C1	Mix C2	Mix C3	Mix C4	Mix C5
0	16.31	12.60	21.73	16.53	10.48
2	16.31	12.60	21.73	16.53	10.48
4	29.15	20.78	20.67	24.97	19.41
